# Enhancing keratinized mucosa around dental implant: Effective techniques and strategies - A review

**DOI:** 10.6026/9732063002001869

**Published:** 2024-12-31

**Authors:** Pavithra Gopalakrishnan, Anitha Logaranjani, Jaideep Mahendra, Pragya G., Aarudra Devi

**Affiliations:** 1 Department of Periodontics, Meenakshi Ammal Dental College and Hospital, Maduravoyal, Chennai, Tamil Nadu, India

**Keywords:** Implant, soft tissue augmentation, keratinized mucosa

## Abstract

Periodontal plastic surgery has been developed around dental implants as implantology has expanded and esthetic demand for replacements has grown. Keratinized mucosa thickness of at least 2mm is recommended to achieve the esthetic appearance and prevent recessions around implant prosthetic rehabilitation. Failure to recognize existing or to anticipate future soft tissue deficiencies may risk the success of dental implants.

## Background:

The introduction of dental implants in dentistry, their popularity and range of applications have exponentially increased. Dental implants are considered the treatment of choice to replace missing teeth for edentulous patients and proven effective based on high survival rates and long-term predictable outcomes. While osseointegration remains the predominant parameter in recognizing the success of dental implants, other parameters related to implant success includes implant fixtures, peri-implant soft tissue, prosthesis and patient satisfaction [1]. Peri-implant tissues are those that surround the osseointegrated dental implants. They are divided into soft and hard tissues. The soft tissue surrounding dental implants is called "peri-implant mucosa" and its characteristics are determined during the healing process following implant placement or the abutment connection. Peri-implant mucosa, like gingiva is covered by a keratinized epithelium that is followed by a thin barrier epithelium, similar to the gingival junctional epithelium that is in direct contact with the abutment; this epithelium continues up to 1 - 1.5 mm coronally to the bone crest [2]. The term keratinized mucosa (KM) defines the external characteristic of the soft tissue between the mucosal margin and the muco-gingival junction. A minimum of 2 mm of KM is considered necessary for peri-implant health, thus facilitating proper oral hygiene procedures [3]. The most commonly encountered soft tissue discrepancies in the anterior zone include facial recession which is related to lack of buccal bone, insufficient papilla height and gingival asymmetry between teeth and implants. Exposed metal or any visible discrepancies in soft tissue volume or margins suggesting an implant-supported prosthesis in anterior regions have become largely unacceptable by patients. In contrast, posterior implants typically present with lack of KM as the predominant soft tissue deficiency. Soft tissue grafting around dental implants has been recommended to enhance functional, biological and esthetic outcomes which varies according to flap design, graft material and suturing technique [4]. The methods and techniques used for gain of keratinized mucosa include vestibuloplasty/apically positioned flap, free gingival graft (FGG), sub epithelial connective tissue graft (SCTG), a cellular dermal matrix (ADM) and xenogeneic bilayer collagen matrix (XCM) [1]. Therefore, it is of interest to review the various techniques to increase keratinized mucosa around implant.

## Keratinized mucosa around implant (KM):

The term "keratinized mucosa" defines the external characteristic of the soft tissue between the mucosal margin and the mucogingival junction; if keratinized tissue is clinically absent, there is only mucosa surrounding implants and abutments [16]. The need of keratinized mucosa adjacent to dental implants is especially important because the implant restoration is located beneath the oral mucosa and it should hide the sub gingival part of the abutment and this can be extrapolated to implant crown restorations [5]. Implants placed in areas lacking keratinized mucosa had higher susceptibility to tissue breakdown than teeth due to plaque accumulation. Despite similar plaque levels, implants placed in no keratinized areas showed earlier loss of attachment [6]. Implants with a narrow zone of keratinized mucosa had a significantly three times higher chance of probing/bleeding (89% vs. 71%) and significantly higher mean alveolar bone loss than implants with a wider zone of keratinized mucosa. Most clinicians prefer to surround the implant with an adequate zone of keratinized mucosa [7]. The advantages include the overall health of the tissues, greater patient satisfaction and fewer complications. As a result of the increased stability of the tissues, prosthetic techniques become more precise [18].

## Various techniques to increase keratinized mucosa around implant:

## Vestibuloplasty (Full Thickness):

Vestibuloplasty is the surgical procedure whereby the oral vestibule is deepened by changing the soft tissue attachments. A mandibular labial Vestibuloplasty combined with lowering of the floor of the mouth was also indicated by MacIntosh RB and Obwegeser HL 1967 to increase the relative height of the residual ridge on the lingual side [8].

## Surgical steps:

In vestibuloplasty, incisions are made bilaterally to the depth of the vestibule on either side of the anterior nasal choanae and down to the periosteum. The mucosae are elevated superiorly to the planned height of the repair. The full-thickness grafts are taken (one from each side of the palate) and each is applied to the exposed periosteum in the canine fossa. No sutures or tacks are required. A prefabricated palatal stent is employed instead. It will affix the grafts with its added labial flanges and will also serve to protect the palatal donor sites when placed over surgical packing. The stent is removed after 5 to 7 days [9].

## Apically repositioned flap:

It is indicated when there is an inadequate band of keratinized tissue in the anticipated buccal area of the dental implant, of at least 2mm; an apically repositioned flap can be employed on the buccal side of the site. Surgical steps begin with an envelope flap design (H design), placing a crestal incision lingual to the cover screw(s) with a minimum 2.0 mm band of keratinized tissue. Displace this tissue apically using two cross arch incisions, which extend vertically avoiding the papilla-like tissue on the proximal side of the adjacent teeth. Elevate the flap to full thickness and apical position it with the edge of the keratinized band buccal of the now exposed implant fixture(s) [10].

## Free gingival graft (FGG):

Free gingival grafts (FGGs) first paved the way for periodontal plastic surgery procedures. Bjorn in 1966 described a technique harvesting auto-genous gingival grafts containing the epithelium and lamina propria to treat areas of the dentition that had loss of attached keratinized tissue due to periodontitis. Indications to increase the band of keratinized tissue around implants and soft tissue augmentation of edentulous ridges [11].

## Surgical steps:

## Recipient site preparation:

A split-thickness flap is prepared along the mucogingival border. The flap design consists of a horizontal incision and two vertical incisions that are elongated to or apically to the mucogingival border depending on the amount of the apical displacement of the partial-thickness flap with 15C or 12D blade [12]. Muscle attachment, loose connective tissue fibres are removed from the periosteal surface. The partial-thickness flap is prepared; the flap is sutured in a new apical position. Sutures must engage the flap and the rigid periosteal surface in order to stabilize the flap. After stabilization, the graft must be completely immobile, intimately adapted to the periosteal surface with no dead space [17].

## Donor site preparation:

The design of the flap consists of four incisions outlining the graft-coronal horizontal incision, mesial and distal vertical incision and apical horizontal incision. Usually, the goal is to harvest an FGG with thickness not exceeding 1.5 mm. For depth orientation during the performance of the outlining incision of the future graft, only the bevelled part of the blade can be used which dimensions is approximately 1 mm. During healing, FGG undergoes contraction of around 30% of initially gain keratinized tissue band. This should be considered while determining the dimension of the graft, which should be 30% larger than the site needing augmentation. The wound in the donor site is protected either by sutures, absorbable gelatine sponge, cyanoacrylate bio adhesive, periodontal dressing, palatal stents, platelet-rich fibrin, or a combination of these techniques [19].

## Sub-epithelial connective tissue graft:

Edel A in 1974 gave the first description of connective tissue graft for increasing the width of gingiva. CTG is still regarded as the gold standard for most soft tissue augmentation treatments. CTG can be divided into two groups. Indications to increase the width of the keratinized gingiva for the treatment of mucosal recession around implants and augmentation of peri-implant soft tissue and the donor preparation (palate) mesiodistally extending from the distal line angle of the canine to the mesial line angle of the palatal root of the first molar. Apically, the donor area is limited with a zone containing blood vessels. The average distance between blood vessels and CEJ of adjacent teeth is 12mm. The recommended apical limit of the donor area is set at 10mm from CEJ, leaving 2 additional millimetres of the safety zone between the apical border of the CTG and the blood vessels [13].

## Surgical steps:

The dissection of the primary flap starts with a horizontal incision 1.0-1.5 mm deep, 2 mm apical from the cement enamel junction and perpendicular to the mucosal surface. The blade angulation is changed to approximately 135°C and a split thickness flap is prepared in the apical direction. The blade is flattened until it becomes parallel with the gingival surface. The dissection is controlled from the external aspect of the flap in order to prevent flap perforation. The partial-thickness flap preparations end after reaching 8mm from the first horizontal incision; this is 10mm apically from the cementoenamel junction, leaving a safe zone with 2mm of distance from the possible location of blood vessels. The primary flap is prepared with the sharp dissection, in a split-thickness manner. During the partial-thickness preparation, the blade is oriented parallel with the mucosal surface to prevent perforations or over thinning of the primary flap. Care must be taken to leave the minimum residual thickness of the primary flap at least 1.5mm, otherwise it could be necrotized. After finishing its dissection, the primary flap is partially reflected and the connective tissue graft is dissected just beneath it. The connective tissue graft can be harvested with or without the periosteum layer, depending on if it is inner surface is prepared with sharp or blunt dissection. The CTG with the periosteum has better mechanical stability and better clinical handling. On the other hand, leaving a periosteal surface on the bone in the donor area will improve the healing of the primary flap. After the completion of the harvesting procedure, the primary flap is repositioned and sutured in its original position. A cross matrass or a combination of parallel and cross sutures is recommended. Advantages of SCTG are bilaminar blood supply is a key component for the predictability of SCTG techniques and the increased root coverage associated with this technique and there is no need for retention of the epithelium. Thus, graft harvesting can be a closed wound procedure that minimizes patient discomfort and risk for bleeding [14].

## Substitutional graft:

## Allogeneic materials:

Compared with autogenously grafts, allografts are readily available. Allogenic a cellular dermal matrix is used for soft tissue reconstruction before bone grafting to reduce the risk of exposure and failure of the bone graft. Various allogeneic grafts are alloDerm®, matrix HD, epiflex®, puros dermis®, AS210 ® and xenogeneic grafts are mucoderm® [15].

## Conclusion:

There are many periodontal plastic surgical techniques that can be used for soft tissue management around dental implants. Surgeons can choose a suitable technique for improving the KM around dental implants not only by increasing the "quality" but also by enhancing the "quantity" to achieve a long-term stable esthetic outcome.
